# Considerations for Swimmers with Type 1 Diabetes: A Narrative Review

**DOI:** 10.3390/sports14100422

**Published:** 2026-10-01

**Authors:** Andrija Djuranovic, Sebastijan Higl, Tijana Cirkovic Higl, Milena Antic, Gordana Korolija Mrdjanov, Djordje Batinic, Milos Maletic, Jovana Ristic, Vladimir Jakovljevic

**Affiliations:** 1 Medical Department, Serbian Institute of Sport and Sports Medicine, 11000 Belgrade, Serbia; milena.antic@rzsport.gov.rs (M.A.); gordana.korolija@rzsport.gov.rs (G.K.M.); djordje.batinic@rzsport.gov.rs (D.B.); milos.maletic@rzsport.gov.rs (M.M.); drvladakgbg@yahoo.com (V.J.); 2 Faculty of Sport and Physical Education, University of Belgrade, 11000 Belgrade, Serbia; sebastijan.higl@fsfv.bg.ac.rs; 3 PR Consulting and Testing TCH, Psychologist and Psychotherapist, 11000 Belgrade, Serbia; tijanacirkovic85@gmail.com; 4 Institute for Cardiovascular Diseases “Dedinje”, 11000 Belgrade, Serbia; jovanaristic00@gmail.com; 5 Department of Physiology, Faculty of Medical Sciences, University of Kragujevac, 34000 Kragujevac, Serbia; 6 Center of Excellence for the Study of Redox Balance in Cardiovascular and Metabolic Disorders, University of Kragujevac, 34000 Kragujevac, Serbia; 7 Department of Pathophysiology, First Moscow State Medical University IM Sechenov, 119435 Moscow, Russia; 8 Faculty of Medicine, University of Banja Luka, 78000 Banja Luka, Bosnia and Herzegovina

**Keywords:** swimming, type 1 diabetes, CGM, carbohydrate, nutrition, diabulimia

## Abstract

Achieving success at the highest level in swimming requires exceptional physical abilities and technical skills. Type 1 diabetes (T1D) poses an additional challenge to maintaining optimal glycemic control. Most information on the nutritional aspects of competitive swimming is available in the literature, but specific considerations for swimmers with T1D are scarce. To excel, swimmers’ nutrition and fueling strategies must align with the varied training and competition requirements, which can elicit different glycemic responses. Continuous glucose monitoring can help develop optimal fueling strategies. Dryland and altitude training will require different approaches than regular pool sessions. Competition-related stress can make glycemia unpredictable, and practiced scenarios must be established. The importance of early recognition of hypoglycemia with planned and practiced treatment scenarios should be established. There is limited evidence on the safety of using ergogenic aids other than caffeine in swimmers with T1D. Given the higher incidence of disordered eating behaviors and eating disorders among swimmers and other individuals with T1D, coaches and everyone involved in the swimmers’ environment should be educated on how to communicate potential body composition issues and, if necessary, manage body composition safely. Swimmers and their team members should be aware of the specific considerations for T1D in swimming for safe sports participation, as presented in this review.

## 1. Introduction

Swimming has been a part of the Olympic Games since 1896. Swimmers can participate in butterfly, breaststroke, backstroke, freestyle, and medley (which combines all four styles in a single race). Pool events range in distance from 50 m to 1500 m, depending on the stroke. Open water competitions vary from 1 to 25 km and beyond, with individual and relay events. Longer distances require hours to compete and include feeding stations along the course. Swimmers can also take part in relay events, either freestyle or medley [1]. Success at the highest level in swimming requires great effort, including technical demands across four strokes and varying distances, as well as specific anthropometric requirements and significant mental effort [2]. Adequate training for the given discipline leads to desired adaptations and the ability to utilize the fuel systems accordingly. The intensity of swimming is the primary factor determining the metabolic pathway required, whether aerobic or anaerobic. Intensity can also be quantified as a percentage of maximal oxygen consumption (VO_2_max), with higher intensity resulting in a greater proportion of CHO utilization and an increased rate of anaerobic metabolism. Anaerobic glycolysis serves as the primary anaerobic energy pathway when the intensity exceeds what can be supplied aerobically (through aerobic glycolysis). The other anaerobic energy pathway, the phosphagen system, generates ATP for brief bursts of activity lasting 8–10 s, which is crucial for short sprints. Understanding the energy systems used in swimming is essential for optimizing fuel intake and aligning training volume and intensity [3].

Comprehensive nutritional guidelines are available for swimmers [4], but no specific guidelines exist for swimmers with type 1 diabetes (T1D). They must carefully optimize CHO intake and insulin dosage and timing to align with their energy expenditure (EE) and training or competition requirements. Monitoring blood glucose level (BGL) throughout the day and maintaining optimal body composition are challenging with T1D [5]. The use of continuous glucose monitors (CGMs) assists with better BGL tracking and implementation of effective fueling strategies. Therefore, this review will examine nutritional strategies and the application of available technologies to improve the health and performance of swimmers with T1D. Given the body image issues present in swimming, the risk of eating disorders (EDs) and disordered eating (DE) will also be addressed. Due to the focus of this review, open-water swimming and other aquatic sports will not be considered. This review mainly targets adolescent and adult competitive and elite swimmers with periodized training and provides information to help nutritionists, coaches, team physicians, and other professionals who work with swimmers with T1D.

## 2. Methodology

The advanced search option in PubMed was used to identify relevant manuscripts up to 31 January 2026. The main terms used were “swimming,” “type 1 diabetes”, “sport,” “CGM,” and “athlete.” The searches yielded few relevant results regarding swimming and T1D, so the search was broadened to include any other sport. The additional search was performed using the same method with the terms “eating disorder,” “disordered eating,” and “diabulimia.” Relevant books on swimming, T1D, and online resources were also consulted. A total of 150 abstracts were assessed for relevance, including abstracts that mention T1D in relation to swimming or T1D in relation to other sports. The list was narrowed to about 70 papers considered relevant to include in the review. Priority was given primarily to papers specific to T1D and swimming, and then to T1D in other sports, along with consensus statements on athletes with T1D.

## 3. Type 1 Diabetes

Diabetes mellitus refers to a group of common metabolic disorders that share the characteristic of hyperglycemia and are classified according to the pathogenic processes that lead to this condition, rather than by earlier criteria such as age of onset or type of treatment [6]. Previously known as juvenile diabetes, T1D is an autoimmune disease in which the immune system destroys the insulin-producing beta cells [7]. Ultimately, the lack of insulin production necessitates constant administration of exogenous insulin. This can be accomplished through intensive insulin replacement using multiple daily injections (MDIs) of insulin or via an insulin pump [8]. The availability of insulin pumps varies by country, and their cost is higher than that of standard injections [8]. Therefore, only MDIs will be discussed, with the understanding that swimming is possible while on CSII.

The MDI plan that offers the greatest flexibility and lowest risk of hypoglycemia combines a long-acting insulin analog (LAA) with a rapid-acting insulin analog (RAA) or an ultra-rapid-acting insulin analog (URAA) [9]. LAA is typically administered once daily and accounts for approximately 30–50% of the total daily dose. Mealtime and correction doses require either URAA or RAA, based on the insulin-to-carb ratio and/or the insulin sensitivity factor, alongside target glucose levels. Potential drawbacks include the need for at least four daily injections. The smallest insulin increment is one unit (or 0.5 units with certain pens). LAAs may not sufficiently address the pronounced dawn phenomenon (the rise in glucose during the early morning hours). Additionally, the price of these insulins may be higher. Other MDI plans are not included in this review because their less flexible approach is not ideal for professional swimmers.

### Insulin Pumps

Insulin can be administered via insulin pumps, as continuous subcutaneous insulin infusion (CSII), or Automated Insulin Delivery (AID) Systems. Insulin is delivered at a basal rate and as a bolus dose. AID systems consist of three components: an insulin pump, a CGM system, and an algorithm that determines insulin delivery. AID systems are preferred over CSII or MDIs because they can adjust the basal insulin rate based on CGM readings [10]. Not all pumps are waterproof, and some must be disconnected while swimming. Others, like the OmniPod © (Insulet Corporation, Acton, MA, USA), are better suited for swimming since they do not involve tubing (an IP28 rating for up to 7.6 m for 60 min) [11,12]. However, the adhesive that secures this pump to the body may loosen in water. In one study [13], a 30 min pump disconnection caused glucose to rise because basal insulin infusion was interrupted, with a rise of about 0.06 mmol/L for each minute the infusion was interrupted. A study by Zaharieva et al. [14] on 7 swimmers with T1D using OmniPod 5 © (Insulet Corporation, Acton, MA, USA) AID showed largely preserved automation in water (from 124 swims lasting 23 to 146 min).

## 4. The Use of Continuous Glucose Monitors

The use of CGMs in patients with T1D has been shown to improve glycemic control and quality of life while minimizing hypoglycemia [8]. CGMs are medical devices that consist of a sensor (inserted into the subcutaneous tissue via a small skin puncture to measure BGL in the interstitial fluid), a transmitter, and a receiver that displays data, usually on a smartphone (modern CGMs support Bluetooth connections) [15]. Compared with traditional blood glucose meters (BGMs), CGMs offer continuous glucose monitoring over a 24 h period, identifying episodes of hyperglycemia and hypoglycemia in real time and tracking glycemic trends. This enables prompt responses to undesirable changes in glucose by adjusting dietary intake and/or insulin dosing. CGMs can last from 3 to 14 days, depending on the type of sensor used [16].

Most CGM systems in use today fall into two categories, real-time CGM (rtCGM) and intermittently scanned CGM (isCGM); the latter is also known as “flash glucose monitoring.” While rtCGM systems generally provide new glucose readings every few minutes, isCGM systems require users to scan the sensor to access current glucose values, either on a dedicated reader or via an app-enabled smartphone [17]. For athletes with T1D, CGM offers advantages over self-monitoring of BGL using handheld BGMs and capillary blood samples. For instance, the ability to monitor BGL in real time during extended aerobic exercise can be used to initiate CHO feeding before hypoglycemia occurs [18]. During pool sessions, the coach or another team member can monitor the swimmers’ glycemia fluctuations on a smartphone and intervene before hypoglycemia occurs. It is important to note that the signal can be interrupted if the smartphone (reader) is too far from the CGM (range varies by brand), so glycemia checks can be performed during breaks.

Exercise appears to compromise the accuracy of both rtCGM and isCGM devices, at least in part due to a substantial lag between interstitial and capillary glucose. rtCGMs often underestimate the decline in glucose levels during exercise [19]. CGMs may also underestimate hyperglycemia, as highlighted in a study examining “high-intensity interval training” sessions, with a time lag of up to 35 min [20]. Consequently, both the type and intensity of physical activity affect the accuracy of rtCGMs. Possible mechanisms influencing accuracy include the rate of glucose change, oxygen concentration, elevated temperature, lactic acidosis, and metabolite variations. Based on CGM data collected over time, swimmers can establish different glycemic targets for various situations. When using CGMs in water, it is important to review product characteristics. For instance, the Freestyle Libre 3 © (Abbott Laboratories, Abbott Park, IL, USA) sensor is water-resistant up to 1 m for as long as 30 min and has a 10 m range [21]. The Dexcom G7 © (Dexcom Inc., San Diego, CA, USA) sensor is waterproof up to 2.4 m for about 24 h and has a 6 m range [22]. According to World Aquatic regulations, the water temperature in indoor pools should be between 25 °C and 28 °C, and open water competitions have temperatures down to 16.0 °C and up to 31 °C [23]. Given the broader operating temperature range of most CGMs and the testing conducted in pure water, CGM readings are expected to remain unaffected. However, factors such as saltwater, saunas, ice baths, and extreme environmental temperatures can influence readings, so swimmers should be aware of this. Protective bandages can also be used and replaced if sessions occur frequently; “dry-land” training can lead to a higher sweat rate and more humid conditions outdoors. Device availability and reimbursement vary by country.

Several other potential issues related to CGMs should be acknowledged. Contact dermatitis (both irritant and allergic) has been documented with all skin-attached devices. It is important to note adhesive reactions, as tape formulations may change over time. Patch testing can sometimes pinpoint the cause of contact dermatitis [10]. Medications such as Paracetamol (Acetaminophen) taken in doses of more than 4 g per day or hydroxyurea and vitamin C in doses exceeding 500 to 1000 mg per day can yield falsely elevated readings on certain sensors [10].

CGMs are a valuable tool for swimmers with T1D to monitor BGL. Technical characteristics should be reviewed, and bandages or tape can be used to keep the sensor in place. If the swimmer is unsure whether the reading is correct, a finger prick should be performed to assess BGL.

## 5. Training and Periodization

### 5.1. Swimming Training Concepts

Competitive swimming races range from about 20 s to 16 min (for the 50 m and 1500 m events, respectively). Swimmers’ performance depends on well-developed technical skills and on both aerobic and anaerobic energy systems. Traditionally, aerobic and anaerobic energy systems have been assessed through VO_2_max testing and blood lactate concentrations. Heart rate serves as an additional indicator in practical settings and is occasionally used independently when more expensive instruments are unavailable [24]. Training zone classification has been used for many years, and terminology varies. Regardless of the terms used, understanding the energy systems involved in different training sessions is important for creating an appropriate fueling and nutrition plan. As summarized by the British Swimming team [25], Table 1 provides a clear overview of various classifications and can improve communication with coaches. It should be noted that glycemic responses described in this table represent tendencies and not certain outcomes, and careful BGL monitoring during various training scenarios should be employed to assess the glycemia trends.

Swimmers require substantial mechanical power output and muscular strength to achieve optimal swimming performance. The ability to generate force in water is crucial for competitive swimming. Coaches implement “dry-land” strength and conditioning programs to enhance athletes’ strength with the objective of improving swimming performance [26]. A variety of training modalities are used, including combined strength and endurance, maximal strength, plyometrics, core-specific, and hypertrophy training. Given the diverse glycemic responses (discussed in detail in the next section), it is essential to tailor the fuel plan and nutrition for these sessions and adjust the insulin dose as needed. Coaches periodize athletes’ training loads across multi-year and annual training programs to achieve optimal competition performance. Periodization is the intentional sequencing of training units (long-, medium-, and short-term training cycles, including microcycles and sessions) designed to elicit adaptations that peak during major competitions. Current theoretical models of annual periodization advocate cyclical or wave variations in training load, progressing from the start of each general training mesocycle toward increasingly specific and intensive overload periods before the taper [27]. Depending on the personalized training plan, various training programs can be employed with regard to changing training volume over weeks [27]. During the taper phase, reducing training volume while maintaining intensity helps enhance adaptations, allowing athletes to recover from the adverse effects of physiological stress. Taper strategies for swimming are discussed in detail elsewhere [28]. It should be noted that for athletes with T1D, the reduction in training volume during taper can change glucose trends [17], and may decrease insulin sensitivity resulting in higher BGL. Different taper strategies, like more low-intensity work and less intense exercise, might be optimal. CHO intake and insulin dosing will need to be adjusted based on the volume and the intensity of the specific microcycle.

**Table 1:** Classification of swimming training and the anticipated glycemic response in swimmers with type 1 diabetes.

Training Zones	British Swimming	Description	HR (bbm) ^a^	Log Book (Simplified)	Sweetenham and Atkinson		Maglischo	Olbrecht	Lactate ^b^ (mmol/L)	Key Energy Source	Training Methods and Pace	Expected Glycemic Response ^c^
1	A1	Aerobic Low Intensity	>50	Aerobic	Zone 1	A1	EN1	AEC	A1: <1	Aerobic: fat	Base conditioning and technical training; warm-up and warm-down; long-duration	↘(decrease)
						A2			A2: ≈1	Aerobic: fat-CHO	Base aerobic training: long-duration exercise with intervals	
	A2	Aerobic Maintenance	40–50			A3	EN2		A3: ≈3		Base aerobic training; >2000 m pace	
2	AT	Anaerobic Threshold	30–40 20–30									↕ (generally stable)
					Zone 2	AT			AT: ≈4	Aerobic and anaerobic: CHO	2000 m pace; MLSS (lactate production = lactate removal)	
3	VO_2_max	Aerobic Overload	10–20	Race Pace	Zone 3	MVO_2_	EN3	AEP	VO_2_max: ≈6–8		Longer duration efforts, medium to low rest; 400 m pace	
4	LP	Lactate Production	0–10		Zone 4	LP	SP2	ANC	LP, LT: >8		High intensity with medium rest; >200 m pace Lactate production: intensity results in the maximal speed of lactate build-up (includes race pace training) Lactate Tolerance: High-intensity work with medium rest to improve buffering	
	LT	Lactate Tolerance	0–10			LT	SP1	ANP				
5	Speed	Basic Speed ATP-PCr	N/A	Race Speed	Zone 5	Sprint	SP3	Sprint	N/A	PCr/ anaerobic: CHO	High intensity, shorter duration with long rest; >50 m pace	↗ (increase) or ↘ (decrease) ^d^

Adapted from: [4,24,25,29]. ^a^ Individual maximum achieved HR should be used to calculate HR. ^b^ Blood lactate values may vary depending on various factors, as mentioned in the text (Section 5.2). ^c^ Considered as tendencies and not certain trends, as high individual variability exists in the blood glucose responses to different forms of exercise. ^d^ Data from other sports show increase in glycemia in this training zone. Data from the Omnipod trial [14] showed that glucose falls regardless of intensity, which should be [14] noted. HR, heart rate; bbm, beats below maximum; A1, Aerobic 1, A2, Aerobic 2; A3, Aerobic 3; LP, Lactate Production; LT, Lactate Tolerance; MVO_2_, VO_2_max maximal oxygen consumption; EN1, Endurance 1; EN2, Endurance 2; EN3, Endurance 3; SP1, Sprint 1; SP2, Sprint 2; SP3, Sprint 3; AEC, aerobic capacity; AEP, aerobic power; ANC, anaerobic capacity; ANP, anaerobic power; MLSS, Maximal Lactate Steady State; CHO, carbohydrate; ATP, adenosine triphosphate; PCr, phosphocreatine; N/A, not applicable.

The social environment, training regimens, and competition impose significant demands on elite athletes, pushing them to the limits of their adaptation. Strategically alternating phases of overload and recovery can minimize performance declines due to injury, overtraining, and detraining while aiming for peak performance during key competitions [30]. Nutritional strategies should be timed and tailored in relation to training volume to support recovery and optimize exercise adaptations [31], particularly for a swimmer with T1D, as inadequate energy and carbohydrate (CHO) intake can lead to undesirable glycemic responses that negatively impact performance. The weekly training program varies depending on the training cycle phase and may include one or two swimming sessions each day, along with “dry-land” training. The training week can also be structured with two or three peaks, where a “peak” is the highest volume and intensity for that week, depending on the schedule and upcoming competitions. Detailed schedule examples and recommendations can be found in the book “Swimming Fastest” by Ernest Maglischo [27]. Understanding the macro-, meso-, and microcycles, along with specific daily schedules, is essential to delivering effective nutritional support.

### 5.2. Lactate Testing

Blood lactate testing is widely used to monitor swimmers’ anaerobic capacities. As explained by Olbrecht [32], blood lactate is influenced not only by the test protocol and the duration and timing of the training session but also by muscle glycogen levels and fatigue. Data on the interpretation of lactate testing in elite athletes with T1D are limited. One study investigated trends in blood lactate levels in 21 adults with T1D on a cycle ergometer [33]. Both tests were performed in the morning, during which the participants consumed a standardized breakfast containing 1 g of CHO/kg body weight (BW) without an insulin bolus, and those using CSII programmed a pump stop. BGL was measured using CGMs. Higher values were observed during the maximal cardiopulmonary exercise test (CPET) than during the 60 min aerobic exercise test at 60% of measured VO_2_max. During CPET, BGL increased with a rise in lactate levels, then stabilized as lactate returned to baseline. During aerobic exercise, some participants exhibited higher BGL immediately after the test. Lactate serves as a substrate for gluconeogenesis in the liver, and elevated lactate levels may reduce insulin’s effect on peripheral glucose uptake, leading to increased BGL [33,34,35].

A study by Romeres et al. used the clamp technique and a lactate tracer to compare lactate turnover in healthy adults and those with T1D [36]. During two separate visits, dextrose, insulin, and lactate infusions were administered and monitored before, during, and after a 60 min exercise test at 65% of measured VO_2_max. Testing was conducted under euglycemic conditions for both groups and under hyperglycemic conditions for the T1D group only. The results indicate that exercising during hyperglycemia inhibits blood lactate clearance, leading to higher blood lactate concentrations during exercise. It also increases the likelihood of reaching the lactate threshold sooner, potentially limiting performance.

Lactate testing can help swimmers with T1D. It should be noted that hyperglycemia can increase lactate levels regardless of high-intensity exercise. Therefore, if blood lactate tests are used, it is reasonable to measure both BGL and lactate levels. Testing should be performed and interpreted with normal BGL (with ketones measured for BGL over 13 mmol/L). Additionally, subjective measures of exercise intensity should be used, especially in cases of high BGL.

## 6. Nutrition, Fueling, and Hydration

### 6.1. Energy Demands of Swimming

The energy cost of moving through water is greater than that of moving on land. EE increases with speed. A swimmer’s physique, including height, limb length, and frontal surface area, can influence the energy cost of movement. Elite swimmers, due to their technical proficiency, exhibit the lowest energy cost compared to recreational swimmers, and this cost decreases as they achieve higher speeds through improved technique [37]. Different strokes yield varying EE, and by accounting for speed, the approximate energy cost can be calculated [38]. In a study evaluating the EE of competitive swimmers [39], freestyle emerged as the most economical, followed by backstroke, butterfly, and breaststroke. A study by Carnero et al. [40] compared 33 young individuals with T1D to healthy controls. Meals were standardized, and physical activity levels were similar between the groups. The 24 h whole-room indirect calorimeter showed significantly higher resting metabolic rate (RMR) and EE in T1D compared with healthy controls (RMR 1708 ± 32 kcal/day, EE 2047 ± 23 kcal/day vs. RMR 1569 ± 34 kcal/day, EE 1908 ± 23 kcal/day). Similar studies in elite athletes are lacking. However, EE does not appear to differ between T1D athletes and non-diabetic athletes. The primary nutritional goal, as with every athlete, is to align energy and CHO intake with EE, considering factors such as training type, intensity, volume, and duration, as well as potential needs for BW and body composition adjustments.

### 6.2. Glycemia, Carbohydrate, and Insulin Optimization

For swimmers with T1D, understanding the glycemic response to different types of training is essential, as it is a key factor in determining BGL during training. Generally, aerobic exercise reduces glycemia, anaerobic exercise increases it, and mixed activities are associated with glucose stability [29]. The anticipated glycemic responses for different swimming training sessions are outlined in Table 1. As noted in the T1D exercise management consensus by Riddell et al. [29], the glycemic response is also affected by the site of insulin delivery, the amount of insulin in circulation, the BGL before exercise, the composition of the last meal or snack, and the intensity and duration of the activity (which determine the percentage of CHO used as fuel in conjunction with circulating insulin). Aerobic exercise typically lowers BGL, although insulin concentrations may not decrease quickly enough at the start of the activity and may rise in the systemic circulation. Incorporating “dry-land” training into the swimmers’ regimen should also be considered, as resistance, circuit, and high-intensity interval training are expected to increase BGL and lessen the decrease in glycemia (as can resistance training done prior to aerobic exercise). Two strategies should be employed to achieve the desired BGL during exercise: optimizing CHO intake (both pre- and during exercise) and administering pre-exercise insulin (or not). However, using exercise type and intensity to manage glycemia (e.g., short sprints to elevate BGL) is not a viable approach for professional swimmers, as failing to adhere to a carefully planned and periodized training program can hinder competitive performance. Still, during warm-up, including sprints (e.g., every fourth length) can be a viable option to prevent the decline in BGL.

One study investigated substrate oxidation in seven physically active individuals with T1D who used CSII during aerobic exercise under euglycemic and hyperglycemic clamps [41]. The results indicate that during exercise, the rate of carbohydrate oxidation was higher in hyperglycemia than in euglycemia. When insulin concentrations were identical, hyperglycemia quickly increased pre-exercise intramuscular glycogen (measured via magnetic resonance spectroscopy), and intramyocellular glycogen breakdown was elevated in hyperglycemia. All of this should be considered when optimizing starting glycemia before training and competition, as well as anticipated substrate utilization at specific intensities. This factor can also impact blood lactate values, which is important to note if lactate testing is used [34,37].

BGL should always be checked before starting a training session. Table 2 presents the optimal starting BGL and provides instructions for proceeding with various BGLs. Section 7 provides specific considerations regarding hypoglycemia and safety.

Based on the recommended CHO intake for athletes [44,45], Table 3 presents optimal CHO periodization and bolus insulin dose corrections for training in professional swimmers. It should be noted that these data are derived from other sports and general T1D recommendations for athletes. Therefore, swimmers can utilize these recommendations as a starting point but will need to closely monitor BGL in various scenarios, and adjust their insulin and CHO accordingly. A reduction in basal insulin may be considered if exercise frequency is high throughout the day or if morning hypoglycemia is frequent. Enhanced insulin sensitivity can persist for 24–48 h after training and can be managed by reducing the bolus insulin dose by about 50% at the meal following exercise, along with a low-glycemic-index snack at bedtime. Late-onset post-exercise hypoglycemia (LOPEH) is particularly likely if training occurs in the afternoon or early evening, increasing the risk of nocturnal hypoglycemia; thus, reductions in both bolus and basal insulin are recommended after training. Hyperglycemia may also occur after intense exercise, especially when insulin levels are excessively lowered [29,46]. Although a “safer” strategy targets a higher starting BGL, excessive CHO intake before sessions can lead to hyperglycemia, necessitating corrective insulin boluses if the BGL becomes too high. Furthermore, hyperinsulinemia has been shown to increase the oxidation of exogenous glucose without sparing muscle glycogen [47], potentially impairing performance. Thus, finding the appropriate carbohydrate–insulin combination for each training session is essential for achieving optimal BGL. Postexercise feeding should primarily focus on CHO intake to replenish muscle glycogen and prevent LOPEH, as noted in Table 3 [29]. This can also be insufficient, as even 48 h postexercise increased insulin sensitivity may persist; there is a real risk of nocturnal hypoglycemia even with adequate refueling after exercise [48]; therefore, a pre-sleep low-GI CHO snack combined with a protein snack may be considered.

Carbohydrate mouth rinse (CMR) can be used during moderate- to high-intensity swimming due to its central mechanism, which reduces fatigue perception [49]. A study comparing the effects of CMR to ingesting a 10% 50 mL CHO solution before each sprint trial on an ergometer indicated a likely beneficial effect from CHO ingestion, while showing no beneficial effect from CMR [50]. Regarding CMR’s effects on insulin and glucose concentrations, a study of eight healthy, trained competitive cyclists administered CMR with 25 mL of a 6.4% glucose solution every 5 km during a 40 km time trial (approximately 1 h total) [51]. Compared with the placebo group, no differences were observed in glucose, insulin, or lactate concentrations at any time point. Additional data are needed to understand how CMR affects glycemia in T1D swimmers. Swimmers can experiment with CMR during more intense training sessions while closely monitoring any potential changes in glycemia. It may also be an effective strategy to minimize gastrointestinal distress that can accompany CHO ingestion.

### 6.3. Macronutrient Planning

Although CHO counting is a fundamental tool for T1D athletes, it does not account for the effects of a balanced meal that includes protein, fat, and dietary fiber. Anticipating how a balanced meal impacts glycemia can be difficult. Both proteins and fats influence glycemia indirectly. In fact, while carbohydrate intake leads to a rapid increase in glycemia within 2–3 h, protein and fat intake elevate glycemia more gradually [52]. Meals higher in fat can increase insulin requirements and lead to late (>3 h) postprandial hyperglycemia [53,54]. Dietary fiber may delay gastric emptying, which can lead to glycemia-reducing effects or at least reduce the rise in BGL [55]. The primary nutrient in fueling for performance is CHO. Protein needs range from 1.2 to 1.6 g/kg BW per day and depend on training type, intensity, and carbohydrate availability. Higher intakes may be necessary for recovery from injury or for individuals on energy-restricted diets who aim to maintain lean body mass. Fat intake can be adjusted based on EE and desired changes in body composition. Macronutrient recommendations are summarized in Table 4. All athletes with T1D should receive counseling and education on the importance of establishing a daily routine for all aspects of diabetes management, with personalized adjustments as needed until an optimal routine is in place. Education should also be provided to everyone working with T1D athletes [56]. Specific data on swimmers with T1D is lacking, and this population should use these numbers as a starting point and modify accordingly.

Mosquera-Lopez et al. [58] used machine learning to develop a statistical model to predict hypoglycemia during and after physical activity. Such models could represent the next advancement in developing personalized, precise guidelines for glycemia management in athletes with T1D.

### 6.4. Altitude Training

Elite swimmers commonly utilize altitude training. They can train at altitude for 2 to 4 weeks, sometimes to prepare for a major competition [59], usually at a moderate altitude of around 2000 m [60]. One study investigated the effect of simulated high altitude (approximately 4300 m) on seven individuals with T1D [61]. Sixty minutes of cycling at 45% VO_2_max at simulated altitude resulted in a significant reduction in BGL at the end of the trial compared to the “sea level” group. This was accompanied by an increased CHO oxidation rate, indicating the impact of acute altitude exposure on higher CHO. Given the potential risk of hypoglycemia and insulin dose adjustments, as in non-T1D athletes, increased CHO oxidation, higher overall energy requirements, and iron status must be taken into account during altitude training camps [62]. Overall, athletes should aim for higher BGLs (e.g., 7–10 mmol/L) to avoid hypoglycemia but avoid exercise if BGLs are high with ketones present to avoid ketoacidosis. Higher CHO intake is expected, given evidence of a higher CHO oxidation rate at altitude. Insulin requirements may be higher for the first 1 to 2 days given the stress that altitude presents, but after acclimatization, basal insulin and bolus should be reduced appropriately.

### 6.5. Hydration

Drinking plain water to thirst and incorporating 6–8% CHO solutions during longer sessions are the primary strategies, aimed at preventing more than a 2% BW loss, which is known to impair performance. A simple way to monitor hydration status is observing urine color. The darker the athlete’s urine, the more concentrated it is and the greater the warning of dehydration, assuming no vitamins or foods impart color [57,63]. Given the low sweat rates at indoor pool temperatures (25–28 °C) and the minimal effect of evaporation in the water, sweat and electrolyte losses are typically small compared with those experienced by “dry-land” athletes [64]. Extra precautions should be taken to ensure optimal hydration during extended outdoor “dry-land” training sessions. One observational study compared self-reported data from athletes with and without T1D [65]. The data showed that fluid intake was greater among T1D athletes. Additionally, 28.9% of T1D athletes in the study reported starting training sessions with BGLs greater than 10 mmol/L. If a swimmer experiences increased thirst and urine production, BGL should be measured because these symptoms can indicate hyperglycemia [66].

## 7. Hypoglycemia and Safety

Swimmers need to recognize the symptoms of hypoglycemia, including dizziness, weakness, headache, hunger, pallor, blurred vision, slurred speech, confusion, irritability, and poor coordination [42]. Sweating is not a reliable indicator during swimming sessions. If hypoglycemia occurs, exercise should stop immediately, and BGL should be monitored every 15 min. Acute hypoglycemia is best treated promptly with 15 g of CHO, such as 1/2 cup of fruit juice, four glucose tablets, 150–200 mL of a sweetened carbonated beverage, or 200 mL of low-fat or skim milk [43]. Severe hypoglycemia should be treated with 1 mg of glucagon administered subcutaneously or intramuscularly to rapidly mobilize liver glycogen, keeping in mind that this treatment is ineffective if liver glycogen stores have been depleted by prolonged, intense exercise [67]. Swimmers should say that they have T1D to other participants in training and/or competitions, if their regular team members are not present, and all team members should familiarize themselves with using glucagon kits. Additionally, it is important to ensure that the glucagon kits have not expired [68]. Athletes may be at an elevated risk of hypoglycemia unawareness during exercise because regular training can blunt the counterregulatory response during hypoglycemia [69].

## 8. Competition

Swimming competitions usually involve a multi-day meet. World Aquatics and Olympic Games programs can last up to 8 days, with heats held in the morning and semifinals and finals in the evening. The overall EE and glycogen depletion during the meet depend on the number of events a swimmer participates in. The more events a swimmer enters, the more challenging it becomes to time fuel maintenance and post-race recovery [4]. Athletes with T1D must take extra precautions to ensure a constant supply of quick-acting CHO snacks, BGMs, insulin pens, and glucagon kits [69]. The main factor present during competition but absent in regular training sessions is stress. Acute stress, such as that experienced in high-level competition, can lead to unpredictable glycemic responses [70]. Given the negative effects of hypoglycemia on performance, athletes should aim to spend less than 1% of their time below target and more than 75% of their time in range (TIR) during competition. In contrast, during the training and preparation phase, TIR should be maintained at 70% [69]. Competition scenarios should be practiced during training.

An important issue is the use of CGMs during competitions. The World Anti-Doping Agency does not address CGM use, and the Australian Institute of Sport has a “No Needles Policy” [71]. Given the growing use of CGMs among non-diabetic athletes, the Union Cycliste Internationale (UCI) has prohibited their use during competitions for athletes without diabetes. However, those with diabetes may use them under medical exception rules, also known as Therapeutic Use Exemptions, which need to be completed prior to any competitions [72]. Therefore, swimmers and their teams should carefully review the current and evolving regulations on CGM use in competitions.

## 9. Caffeine as an Ergogenic Supplement

Athletes commonly use ergogenic supplements to enhance performance, but evidence supporting their use in athletes with T1D is limited. In athletes without T1D, caffeine intake at approximately 3–6 mg/kg body mass, taken 60 min before exercise, appears to yield the most consistent positive effects on sports performance [73]. One study examined the effect of a 6 mg/kg body mass caffeine dose in individuals with T1D during 45 min of steady-state activity at 65–70% VO_2_max [74]. The study showed a reduction in the decrease in glycemia during exercise, although there was a potential risk of LOPEH. Another study [75] showed that combining 250 mg of caffeine with 10 g of glucose reduced exercise-related hypoglycemia. These findings should also be interpreted in light of the fact that ingesting caffeine with higher starting BGL may elevate BGL further, keeping in mind the unpredictability of the response of caffeine on BGL, Also, caffeine can hinder sleep in some individuals, and in those with T1D, sleep disturbances may reduce insulin sensitivity [76]. Swimmers with T1D trialing caffeine use should start with lower dosages and carefully monitor their BGL and sleep.

Other ergogenic aids that may enhance performance [77], including creatine, beta-alanine, and concentrated dietary nitrate, remain uninvestigated for safety in T1D athletes.

## 10. Body Image in Swimming

The swimmers train and compete in swimsuits that can facilitate unhealthy comparisons of body composition and contribute to a distorted body image. The unhealthy practices aimed at achieving a “desired” body composition can lead to DE and EDs [78]. On the other hand, patients with T1D show a higher incidence of DE and EDs. Data vary, but studies indicate that 10–27% of adolescent females and about 8.6% of adolescent males exhibit some form practice on the DE to EDs spectrum [79]. Athletes participating in “lean sports” (per authors, sports that inherently promote a drive for leanness, including aesthetic, endurance, anti-gravitational, and weight-class sports) were shown to be at an elevated risk for DE compared with “non-lean sports” [80]. A 2025 meta-analysis reported an ED prevalence of 27.56% in aquatic athletes [81].

EDs and DE in individuals with T1D manifest as food restriction, binge eating and vomiting, laxative use, and excessive exercise, which can occur with or without insulin misuse. The term “diabulimia” is often used to describe EDs related to T1D when insulin is intentionally taken in inadequate amounts (smaller doses or completely omitted) to control (reduce) BW [82]. However, “diabulimia” is an inaccurate term because the patterns of EDs and DE that manifest are not solely bulimia. If these behaviors persist over time, they can lead to a combination of T1D-related complications (physiological changes, neuropathy, kidney disease, heart disease, eye disease) and various ED complications (loss of bone density, hypotension, hypothermia, bradycardia, peripheral edema, hypertrophy of the salivary glands, dental enamel erosion, etc.) [83]. It is paramount that everyone in the athlete’s environment understands the significant risks associated with combining swimming and EDs. From a young age, appropriate language should be used and suitable practices taught when educating swimmers about safely adjusting their BW and body composition, if necessary. The Swimming Australia team [84] has developed a guide as part of the “Performing in Lycra initiative” to provide the swimming community with educational resources, guidance, and recommendations for establishing appropriate language and safe environments. This document should be reviewed carefully, especially when working with T1D athletes. Additionally, a discussion should take place about the potential stress that may arise from using CGMs.

It is important to screen for Relative Energy Deficiency in Sport (REDs), using the clinical assessment tool (REDs-CAT2) in order to timely prevent and treat REDs [85]. It should be noted that, compared to dry-land sports, stress fractures are not expected to occur in swimmers, but low bone mineral density may still occur.

## 11. Conclusions

**Figure 1:**
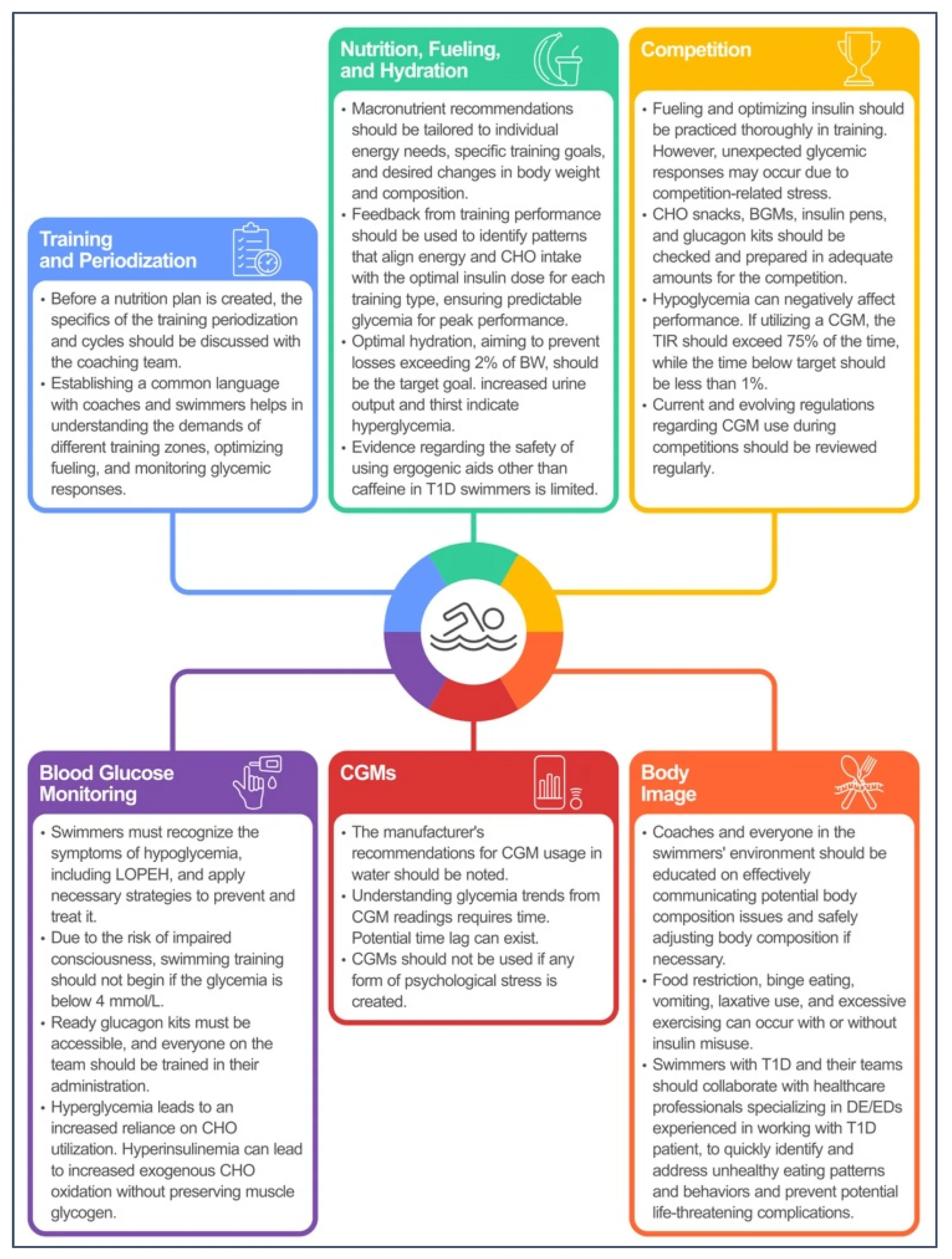
Considerations for swimmers with type 1 diabetes. T1D, type 1 diabetes; Eds, eating disorders; DE, disordered eating; CHO, carbohydrate; BW, body weight; CGMs, continuous glucose monitors; LOPEH, late-onset post-exercise hypoglycemia.

High-level competitive swimming requires precise training periodization and strict adherence to the training plan. To meet the intense demands of the season, it is essential to establish optimal nutrition and fueling strategies. Swimmers with T1D need to maintain optimal BGL to ensure safe and effective training and competition performance. The primary strategies involve practicing CHO fueling and optimizing insulin doses to identify the most effective and predictable combinations and fueling patterns for specific training sessions and races. CGMs are useful tools for tracking BGL, but it is important to acknowledge their limitations. The swimmers’ teams should collaborate closely with healthcare professionals to ensure athlete safety and well-being and to prevent potential EDs from arising in a timely manner. However, evidence in swimmers with T1D is limited, and the data presented in this review combine studies in T1D swimmers with available evidence from athletes with T1D in other sports and consensus statements. Further research is needed in elite swimmers, including randomized intervention studies across various fueling and training scenarios, using CGMs to support training protocols, and investigating how to effectively identify and address EDs, DE and REDs in this population. Machine learning algorithms are being developed to predict glycemic trends and mitigate the risk of hypoglycemia or LOPEH, thereby improving the health and performance of swimmers with T1D. The key advice for swimmers with T1D is presented in Figure 1.

## Figures and Tables

**Table 2:** Blood glucose level just prior to exercise and recommended glucose management strategies for type 1 diabetes swimmers.

Starting Glycemia	Glucose Before Exercise	Practical Advice	Comment
Level 2 hypoglycemia (≤3 mmol/L) and Level 3 (assistance required to manage hypoglycemia irrespective of glycemia)within the previous 24 h	As per team member or healthcare professional intervention (ora CHO). Glucagon kit if the athlete is confused and/or cannot swallow safely, unconscious, or having a seizure	Glucagon ^a,b^ preparations a that do not have to be reconstituted are preferred (in a prefilled pen and a prefilled syringe)	Contraindication to exercise in the 24 h following a severe hypoglycemia episode ^c^
Level 1 hypoglycemia (BGL 3.1–3.9 mmol/L) with the ability to self-treat	Ingest at least 20 g of glucose	Examples of food with about 15 g of fast-acting CHO: 250 mL of isotonic drinks, one small box of juice, half a banana, one glucose gel, one energy bar (check the label)	Vigilance around monitoring; Swimming unsupervised should be avoided
Below target (4–4.9 mmol/L)	Ingest 10–20 g of glucose before starting exercise.		Delay exercise until blood glucose is more than 5 mmol/L and monitor closely for hypoglycemia
Near target (5–6.9 mmol/L)	Ingest 10 g of glucose before starting aerobic exercise		Anaerobic exercise and high-intensity interval training sessions can be started
At target levels (7–10 mmol/L)	/	Fast-acting CHO foods should always be available during training	Aerobic exercise can be started. Anaerobic exercise and high-intensity interval training sessions can be started; glucose concentrations could rise.
Starting BGL slightly above target (10.1–15.0 mmol/L)	/	Fast-acting CHO foods should always be available during training	Aerobic exercise can be started Anaerobic exercise can be started, but glucose concentrations could rise. Monitor glycemia.
Starting BGL above target (>15 mmol/L)	/	/	If the hyperglycemia is unexplained (not associated with a recent meal), check blood ketones. Exercise is contraindicated if ketones are >1.4 mmol/L in blood or ≥2.0 mmol/L in urine

Adapted from: [29,42,43]. ^a^ all team members should be aware of the location of glucagon kits and be educated on how to administer them; ^b^ this treatment is ineffective if liver glycogen stores have been depleted by prolonged, intense exercise. ^c^ If a hypoglycemia episode occurs in the morning session, with appropriate refueling and recovery afterwards, stable BGL during the day and no symptoms, evening session may be considered. *T1D*, type 1 diabetes mellitus, *BGL*, blood glucose level, *CHO*, carbohydrate.

**Table 3:** Periodization of carbohydrate intake and insulin dose adjustments in swimmers with type 1 diabetes.

CHO ^a^		Bolus Insulin Reduction (Training Sessions Lasting 30 min or Longer) ^e,f^
Daily Intake	Around Training Sessions	
**1 training session per day:** **3–7 g/kg BW/day** - higher-end range for days with longer and more intense sessions - lower-end during recovery sessions and taper **≥2 training sessions per day:** **6–10 g/kg BW/day** - higher-end range for days with longer and more intense sessions - lower-end during recovery sessions and taper **1–4 g/kg BW** around the key, high-intensity sessions **0.8–1.2 g/kg BW** immediately after sessions ^b^ to restore muscle glycogen faster, with ≥2 training sessions per day	**Session focus ^c^:** **Skill, AEC, AEP** Mouth rinse; smaller amounts of CHO **30–60 g/h**during **longer** sessions (≥60 min) ^d^	**Skill, AEC:** Reduce 25–50% (greater reduction for longer sessions)
		**AEP, ANC:** Reduce 50–75% (greater reduction for longer sessions)
	**Session focus ^c^:** **AEP, ANC, ANP** **Adequate fueling in 2 h before the session ** **(1–4 g/kg BM)** Mouth rinse; smaller amounts of CHO **30–60 g/h** during **longer** sessions (≥60 min) ^d^	
		**ANC, ANP:** Reduce 75% (greater reduction for longer sessions)
	**Session focus ^c^:** **ANP,** **Sprint** **Adequate fueling in 2 h before the session ** **(1–4 g/kg BW)** Mouth rinse; smaller amounts of CHO	
		**Sprint, resistance training, circuit training:** no reduction recommended

Adapted from: [25,29,44,45]. ^a^ A high glycemic index CHO source should always be available during training in case of hypoglycemia. ^b^ Useful to reduce the risk of hypoglycemia and improve recovery; might need a specified bolus insulin dose depending on the length and intensity of exercise (e.g., reduced insulin to carbohydrate ratio). ^c^ See Table 1 for training swimming training classification. ^d^ High CHO consumption might cause gastric upset in some individuals and contribute to hyperglycemia during and after the activity. Sports drinks containing glucose and fructose might be preferable to increase CHO absorption during exercise and maintain hydration status. ^e^ Suggested reductions in bolus insulin dose before exercise, based on the intensity of exercise, for exercise started within 90 min of consumption of the meal. ^f^ Differences in the site and depth of insulin injection affect absorption characteristics. *CHO*, carbohydrate, *BW*, body weight, *AEC*, aerobic capacity, *AEP*, aerobic power, *ANC*, anaerobic capacity, *ANP*, anaerobic power.

**Table 4:** Recommended macronutrient and fibre intake and food choices for swimmers with type 1 diabetes.

Macronutrient	Recommended Intake ^a^	Food Ideas
**CHO**	Refer to Table 3.	**For the main meals (opting for lower GI CHO will result in more stable glycemia):** - oats with yogurt, berries, and seeds - whole grain: rice, pasta, tortilla - potatoes, sweet potatoes - beans, lentils, soy (+ high-quality protein and fat sources with vegetables) **Between-session snacks or smaller meals:** - toast with jam - fruit like bananas and dates - low-fat yogurt with berries and/or honey - white pasta or rice (smaller portion) **During longer sessions (high GI CHO):** - bananas - energy gels - carbohydrate drinks (6–8% CHO)
**Protein**	**1.2–1.6 g/kg BW/day ** **Spread regularly: 0.3–0.4 g/kg BW throughout the day** **Effect on glycemia: ** **For meals containing at least 30 g of CHO and 40 g of protein, increasing pre-meal insulin by 15–20% should be considered**	**For the main meals:** - high-quality protein sources: meat, fish, seafood, eggs - add dairy products **For recovery:** - **Milk-based drinks containing CHO and protein can assist with recovery after exercise and prevent LOPEH** - whey isolate or shakes (fruit + whey) - protein bars with fruit or carbohydrate drinks
**Fats**	**As needed for energy demands and BW, and body composition goals ** **Effect on glycemia: ** **For high-fat meals (>40 g), an additional insulin bolus should be considered 1 h after the meal, 30–35% pre-meal dose**	**For the main meals (high-quality fats from natural sources)**: - extra virgin olive oil, olives, avocados, cold-pressed coconut oil, organic or grass-fed butter - fish instead of meat (balanced throughout the week) - seeds (i.e., flax, chia) to the morning oatmeal **Snacks**: - nuts (i.e., walnuts, almonds, cashews)
**Dietary fibre**	**15 g of fibre for 1000 kcal ** **Effect on glycemia: ** **Attenuates the rise in BGL**	**Fiber-rich foods should be emphasised, mostly for main meals (ensuring glycemia stability while providing the optimal micronutrient intake):** - whole, fiber-rich grains (quinoa, brown rice, barley, multigrain or black bean pasta, sprouted or whole grain bread, oatmeal, multi or whole grain cereals, and legumes), fruits, vegetables, nuts, seeds

Adapted from: [4,29,46,54,55,57]. ^a^ Specific data on swimmers with T1D is lacking, and this population should use these numbers as a starting point and modify accordingly. *CHO*, carbohydrate; *GI*, glycemic index; *BW*, body weight, *BGL*, blood glucose level, *LOPEH*, late-onset post-exercise hypoglycemia.

## Data Availability

No new data were created or analyzed in this study. Data sharing is not applicable to this article.

## Abbreviations

The following abbreviations are used in this manuscript:

A1	Aerobic 1
A2	Aerobic 2
A3	Aerobic 3
AEC	aerobic capacity
AEP	aerobic power
AID	Automated Insulin Delivery
ANC	anaerobic capacity
ANP	anaerobic power
ATP	adenosine triphosphate
bbm	beats below maximum
BGL	blood glucose level
BGLs	blood glucose levels
BGMs	blood glucose meters
BW	body weight
CHO	carbohydrate
CGM	continuous glucose monitor
CGMs	continuous glucose monitors
CMR	carbohydrate mouth rinse
CPET	cardiopulmonary exercise test
CSII	continuous subcutaneous insulin infusion
DE	disordered eating
EDs	eating disorders
EE	energy expenditure
EN1	Endurance 1
EN2	Endurance 2
EN3	Endurance 3
HR	heart rate
isCGM	intermittently scanned continuous glucose monitor
LAA	long-acting insulin analog
LOPEH	late-onset post-exercise hypoglycemia
LP	Lactate Production
LT	Lactate Tolerance
MDI	multiple daily injections
MLSS	Maximal Lactate Steady State
MVO_2_, VO_2_max	maximal oxygen consumption
N/A	not applicable
PCr	phosphocreatine
SP1	Sprint 1
SP2	Sprint 2
SP3	Sprint 3
RAA	rapid-acting insulin analog
REDs	Relative Energy Deficiency in Sport
REDs CAT2	Relative Energy Deficiency in Sport Clinical Assessment Tool 2
rtCGM	real-time continuous glucose monitor
T1D	Type 1 diabetes
TIR	time in range
URAA	ultra-rapid-acting insulin analog
